# A Step Forward for the Treatment of Localized Prostate Cancer Using Gold Nanoparticles Combined with Laser Irradiation

**DOI:** 10.3390/ijms25084488

**Published:** 2024-04-19

**Authors:** Sara Pinho, Tânia Ferreira-Gonçalves, Joana Lopes, Mariana Neves Amaral, Ana S. Viana, João M. P. Coelho, Maria Manuela Gaspar, Catarina Pinto Reis

**Affiliations:** 1 Research Institute for Medicines, iMed.ULisboa—Faculty of Pharmacy, Universidade de Lisboa, Av. Professor Gama Pinto, 1649-003 Lisboa, Portugal; sara.pinhoo10@gmail.com (S.P.); taniag1@ff.ulisboa.pt (T.F.-G.); joanamargaridalopes@campus.ul.pt (J.L.); marianaamaral@edu.ulisboa.pt (M.N.A.); 2 Instituto de Biofísica e Engenharia Biomédica (IBEB), Faculdade de Ciências, Universidade de Lisboa, Campo Grande, 1749-016 Lisboa, Portugal; jmcoelho@ciencias.ulisboa.pt; 3 Centro de Química Estrutural, Faculdade de Ciências, Universidade de Lisboa, Campo Grande, 1749-016 Lisboa, Portugal; apsemedo@fc.ul.pt

**Keywords:** prostate cancer, photothermal therapy, gold nanoparticles, synthesis, characterization

## Abstract

Prostate cancer (PCA) is the second most common cancer diagnosis in men and the fifth leading cause of death worldwide. The conventional treatments available are beneficial to only a few patients and, in those, some present adverse side effects that eventually affect the quality of life of most patients. Thus, there is an urgent need for effective, less invasive and targeted specific treatments for PCA. Photothermal therapy (PTT) is a minimally invasive therapy that provides a localized effect for tumour cell ablation by activating photothermal agents (PTA) that mediate the conversion of the light beam’s energy into heat at the site. As tumours are unable to easily dissipate heat, they become more susceptible to temperature increases. In the PTT field, gold nanoparticles (AuNPs) have been attracting interest as PTA. The aim of this study was to formulate AuNPs capable of remaining retained in the tumour and subsequently generating heat at the tumour site. AuNPs were synthesized and characterized in terms of size, polydispersity index (PdI), zeta potential (ZP), morphology and the surface plasmon resonance (SPR). The safety of AuNPs and their efficacy were assessed using in vitro models. A preliminary in vivo safety assessment of AuNPs with a mean size lower than 200 nm was confirmed. The morphology was spherical-like and the SPR band showed good absorbance at the laser wavelength. Without laser, AuNPs proved to be safe both in vitro (>70% viability) and in vivo. In addition, with laser irradiation, they proved to be relatively effective in PCA cells. Overall, the formulation appears to be promising for use in PTT.

## 1. Introduction

PCA has the second highest incidence and the fifth highest mortality rate worldwide [1], representing a major impact on men’s health. GLOBOCAN 2020 data predict an increase in the incidence of PCA with 1,017,712 newly diagnosed cases worldwide (+79.7% overall change) [2]; efforts have been made to increase early detection [3] as well as search for innovative treatments able to enhance cancer management, thus improving patients’ quality of life with fewer adverse effects [4,5]. Despite the existence of various therapeutic strategies such as surgery and radiotherapy, they are associated with a lack of specificity towards cancer cells and a wide range of adverse effects that have an impact on the patient’s life [6,7]. Considering the points mentioned above, it is important to look for new, effective and minimally invasive treatments to combat PCA.

In this sense, PTT is a minimally invasive and accurate method that has shown promise in the treatment of localized PCA with a favourable safety profile and rapid recovery time [4,8,9,10]. The heat generated must be sufficient to destroy tumour cells that are more sensitive to temperature increases due to their lower capacity to dissipate heat. On the other hand, the heat must not be excessive such as it results in resistance by heat shock proteins [11,12,13,14,15,16]. The effectiveness of PTT depends on the ability of the light to reach the tumour tissue, since the tissues interact with the light depending on its wavelength [17,18].

In the 650–900 nm biological window [19,20], including light within the Near-Infrared (NIR) range, the tissues have lower absorption, allowing the irradiated light to reach deeper tissues [20,21,22]. The maximum depth described in the literature is around 4–10 mm [23]. The localized effect of the temperature increase in PTT is an interesting point because it can reduce the undesirable effects of the therapy on healthy tissues [12]. However, to improve the effectiveness of PTT, PTA can be used to enhance the heat generated at the tumour site [24,25]. Itis based on the absorption of light energy by PTAs, which accumulate in the tumour and convert the irradiated light into heat, causing hyperthermia of the cells (>41 °C) and consequently their destruction [10,26]. It seems that the mechanism of cell death depends on the wavelength, exposure time and intensity of the radiation, the characteristics of the PTA and their concentration in the tumour microenvironment. [24,25]. Due to the combined action of PTAs and light, there are suggested mechanisms for cell death such as necrosis, apoptosis and oxidative stress. Normally, cell death occurs through necrosis at high temperatures (55 °C) and there is a release of intracellular content into the extracellular microenvironment, which triggers an immune and anti-inflammatory response. On the other hand, apoptosis occurs at relatively lower temperatures (41–47 °C) and signalling processes are triggered. These processes attract dendritic cells and macrophages that “uptake” the damaged cells. This can be followed by secondary necrosis which might trigger inflammatory responses if this process does not take place in a timely manner [24,25,27]. However, the apoptotic route is preferable in vivo because the necrotic route can induce severe secondary inflammation and can also lead to the development of metastases by rupturing the membrane and releasing intracellular content [25,28]. Another mechanism that might occur is the formation of reactive oxygen species (ROS) leading to damage of DNA, proteins, cell membranes and other cellular contents. Subsequently, cellular self-protection mechanisms are activated, namely autophagy, resulting in the uptake of damaged cellular contents into vacuoles which are then degraded by lysosomal enzymes [29].

Gold has been exploited throughout the history of civilization for its assumed properties. Initially in the pharmacopoeia of the 17th century, it was used by Nicholas Culpepper to treat certain illnesses. In the 19th century, gold complexes were used to treat rheumatoid arthritis, and since then it has also been used to treat rheumatic diseases [30] and others [31]. AuNPs and gold-based nanodevices are well documented, and several are in pre-clinical trials in the field of cancer, although they have also been reported in the treatment of other diseases such as HIV, Parkinson’s, ophthalmologic and diabetes [32]. AuNPs are also of potential interest for their anti-inflammatory and antioxidant properties in the treatment of autoimmune and inflammatory diseases, particularly in rheumatoid arthritis [33].

AuNPs have proved to be promising PTAs due to their ability to convert energy into heat through their optical characteristics, such as SPR, which occurs in the presence of light at certain wavelengths [34,35,36]. In addition to this characteristic, AuNPs have low toxicity and they can be functionalized via conjugation with targeting moieties [17,20,37,38]. Their properties depend on the physicochemical characteristics of the AuNPs, such as their size, shape and surface charge [20,39]. Among the methods already proposed for synthesizing AuNPs, the Turkevich method is one the most common. It generally includes cetyl trimethyl ammonium bromide (CTAB), commonly classified as a toxic agent. In this area, several efforts have been made in order to find safer and more environmentally and biologically friendly reducing agents [9,40].

This work focuses on the synthesis and characterization of AuNPs. AuNPs were synthesized according to the protocol already established by our research group [41]. The AuNPs must have appropriate characteristics to be retained in the tumour and not reach healthy tissue [24]. In addition, properties such as size, shape and surface charge influence the optical properties of AuNPs and can also alter their biological effects [20,39]. A physicochemical characterization was performed in terms of average particle size, PdI, ZP, maximum absorption peak and morphology. The safety of AuNPs was assessed in a brine shrimp model, and the efficacy was assessed through thermal activation using in vitro phantoms and also in human PCA cell line (PC-3 cells). The last experiment was performed with a 3-(4,5-dimethylthiazol-2-yl)-2,5-diphenyltetrazolium bromide (MTT) assay.

## 2. Results

### 2.1. Synthesis and Characterization of AuNPs

#### 2.1.1. Physicochemical Characterization

**Figure 1:**
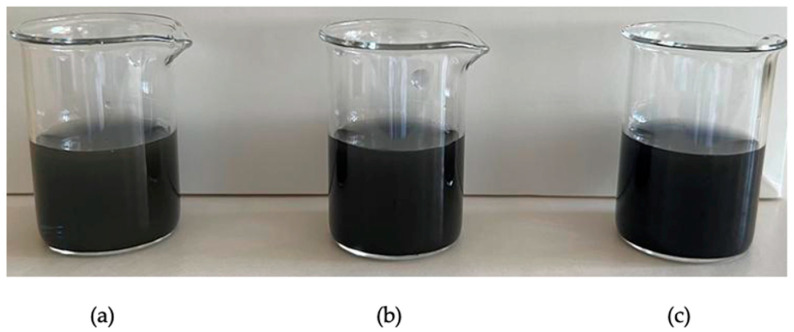
Synthesis of AuNPs before the centrifugation process of three independent batches (**a**–**c**).

AuNPs were successfully produced. Figure 1 depicts the macroscopic aspect of the AuNPs prepared. The diameters of AuNPs were determined by Dynamic Light Scattering (DLS), and the results are presented in Table 1, with average particle size of ca. 100 nm.

**Figure 2:**
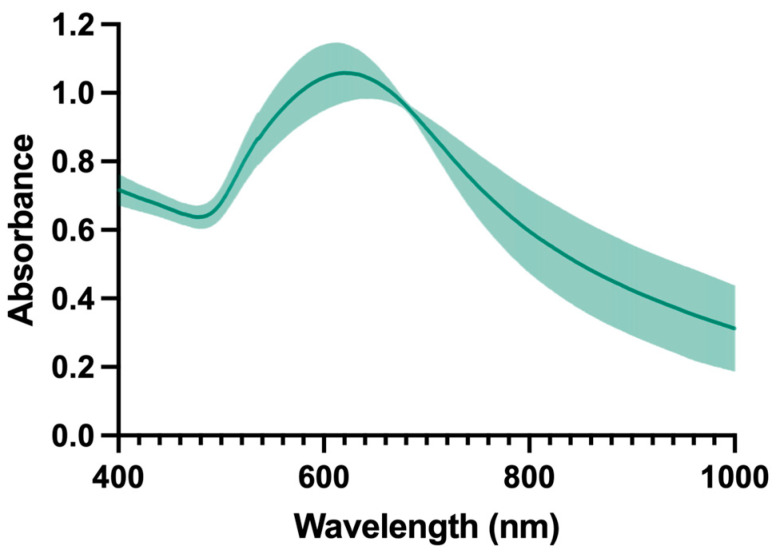
Absorbance of the formulation. Data represented as mean ± SD, n = 3.

Regarding size, the mean size of replicates was around 100 nm with PdI values below 0.15. AuNPs suspended in PBS revealed a negative surface charge and showed absorption within the visible range even though it is close to the optical therapeutic window range (λ ≈ 650–900 nm). Furthermore, there is no narrow peak centred on the maximum value, but there is a broad peak demonstrating high absorbance at wavelengths in the optical therapeutic window (λ ≈ 650–900 nm), as shown in Figure 2. At the specific wavelength of the NIR laser, 808 nm, the batches presented an average absorbance of 0.579.

#### 2.1.2. Morphological Analysis

**Figure 3:**
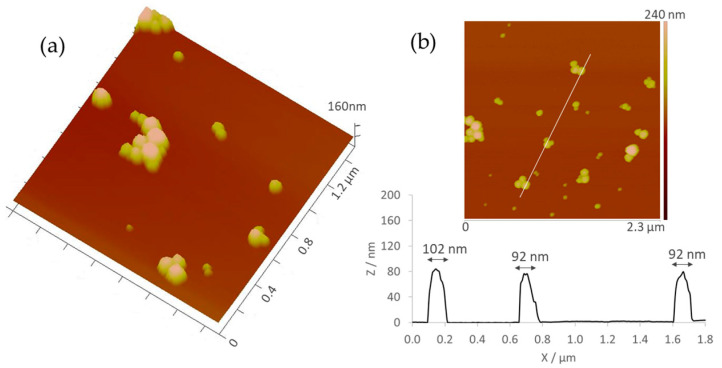
AFM topographical images of AuNPs deposited on mica shown in the (**a**) three-dimensional and (**b**) cross-sectional views.

The morphology of AuNPs was assessed by Atomic Force Microscopy (AFM). All samples showed spherical or quasi-spherical structures as depicted in Figure 3.

### 2.2. Efficacy Assessment in a Phantom Model

**Figure 4:**
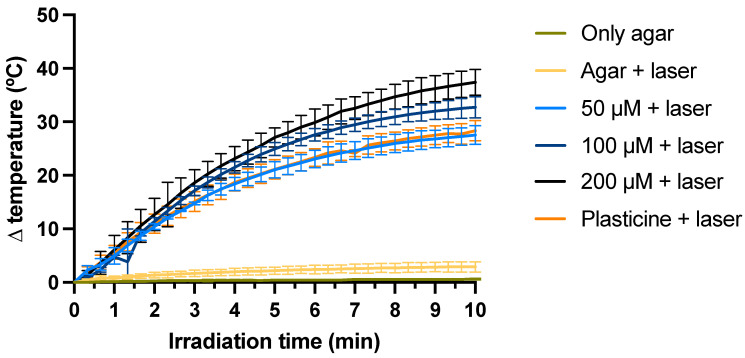
Evolution of the phantom’s temperature as a function of the irradiation time with a NIR laser, for 10 min. The Au concentration was 50, 100 and 200 μM, the same as for the in vitro studies. The results represent the mean value ± SD, n = 3.

The thermal activation of AuNPs by NIR laser was evaluated using an agar phantom model where NPs were incorporated. In this assay, the temperature was recorded before and after irradiation (Figure 4). Agar-only phantoms were used to control possible temperature fluctuations caused by temperature variations in the laboratory. However, no significant changes were detected. Subsequently, those same phantoms were irradiated with laser, being used as controls of the temperature increase caused by irradiation alone (due to direct heating of the thermocouple material). A temperature increase of approximately 3 °C was detected. The phantoms containing a plasticine sphere, used as positive controls, showed a temperature increase of about 28 °C. This increase is because black plasticine strongly absorbs radiation.

For AuNPs in the agar phantoms at a concentration of 50 μM, the temperature rise was identical to the positive control. On the other hand, for phantoms containing AuNPs at a concentration of 100 and 200 μM, increases of 31 and 35 °C, respectively, were observed. Therefore, the temperature variation during 10 min of irradiation increased with increasing AuNP concentration.

**Figure 5:**
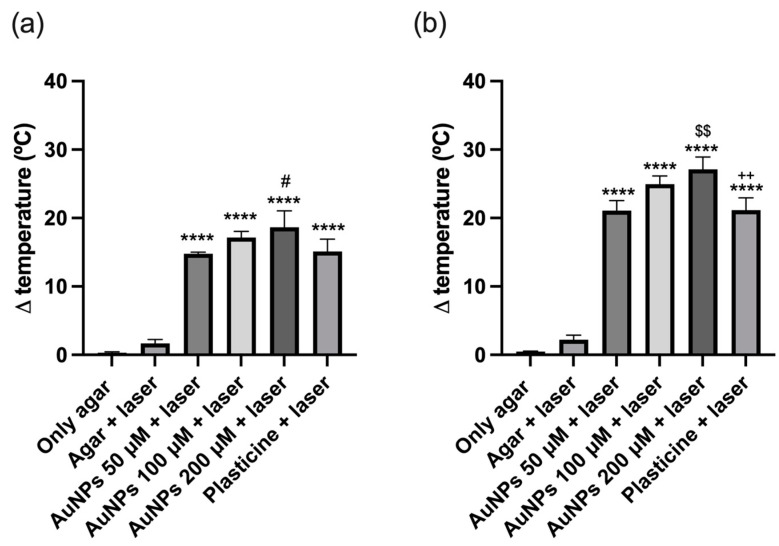
Variation of the temperature of the phantom model upon irradiation with a NIR laser (**a**) for 3 min and (**b**) for 5 min. The results represent the mean value ± SD, n = 3. **** *p* < 0.0001 compared with only agar and agar + laser; ^#^ *p* < 0.032 compared with 50 μM + laser; ^$$^ *p* < 0.032 compared with AuNPs 50 μM + laser; ^++^ *p* < 0.032 compared with AuNPs 200 μM + laser.

The evolution of the phantoms’ temperature up to 10 min is depicted in Figure 4, and the variation of temperature after 3 min and 5 min of irradiation are showed in Figure 5a,b, respectively.

The phantom with AuNPs at 50 μM showed a similar increase relative to the phantom containing the black plasticine sphere: about 15 and 20 °C, at 3 and 5 min after irradiation, respectively. On the other hand, at the higher concentrations (100 and 200 μM), the temperature increase was AuNP concentration-dependent. Thus, at 3 min, the temperature variation was 17 °C when irradiating the cuvettes with 100 μM of AuNPs and 18 °C when they contained 200 μM of AuNPs. At 5 min, we found agreement with the previous results, noticing an increase of 25 and 27 °C, respectively, as we can see in Figure 5.

### 2.3. In Vitro Safety Assessment without Laser

**Figure 6:**
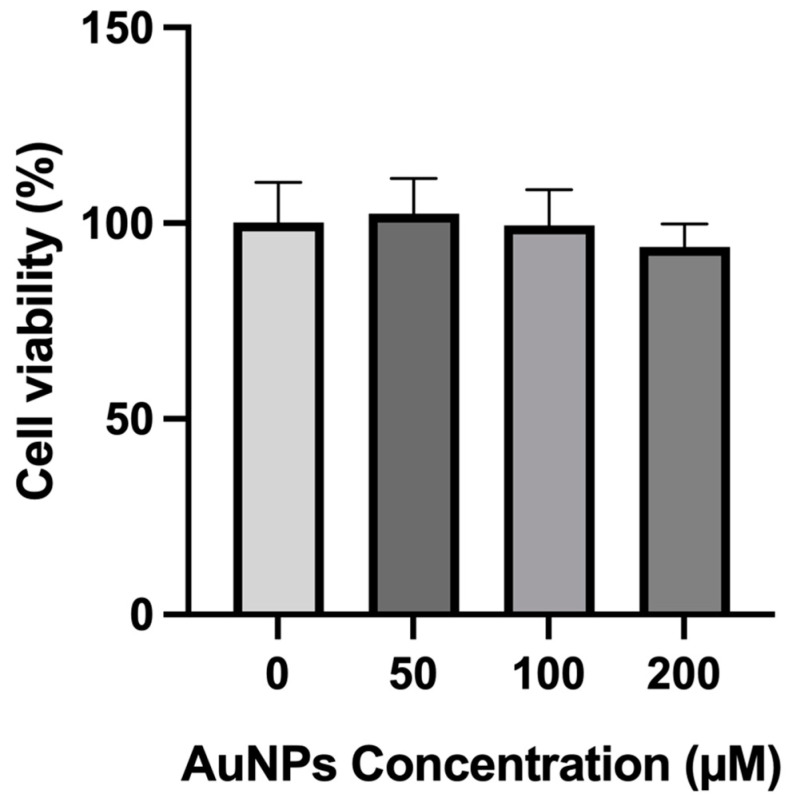
Cell viability (%) of PC-3 cells incubated for 24 h with various concentrations of AuNPs. Untreated cells were used as controls. The results represent the mean value ± SD, n ≥ 5.

The safety of the AuNPs was also in vitro assessed, 24 h after incubation in PC-3 cells. The length of the cell cycle can vary greatly depending on the type of cell. In rapidly dividing cells such as cancer cells, the total length of the cell cycle in mammalian cells is around 24 h, with the S phase typically lasting around 10–12 h, G1 phase around 8–10 h, G2 phase around 4–6 h and M phase (including mitosis and cytokinesis) around 1 h. AuNPs were tested at 3 different concentrations: 50, 100 and 200 μM. The viability was assessed using an MTT assay (Figure 6). Without laser, none of the AuNP concentrations reduced the cell viability. Moreover, there was no statistical significance between the wells with AuNPs at different concentrations and/or the wells with only cells.

### 2.4. Efficacy Assessment in Human PC-3 Cells

Taking into account that the highest variation of temperature occurred with AuNPs at 200 μM, the efficacy at this concentration was also tested in PC-3 cells. Four hours after incubation with AuNPs in PC-3 cells, samples were submitted to irradiation with an 808 nm NIR laser to activate the AuNPs. Two irradiances were tested, 7.7 and 9.4 W/cm^2^, for 3 and 5 min, respectively. In order to study the effectiveness of the formulation, cells with and without AuNPs were also irradiated. In addition, to ensure that the effect of the AuNPs was not due to the laser alone but to an additive or a synergistic effect, cells without AuNPs were also irradiated. Subsequently, cell viability was determined.

**Figure 7:**
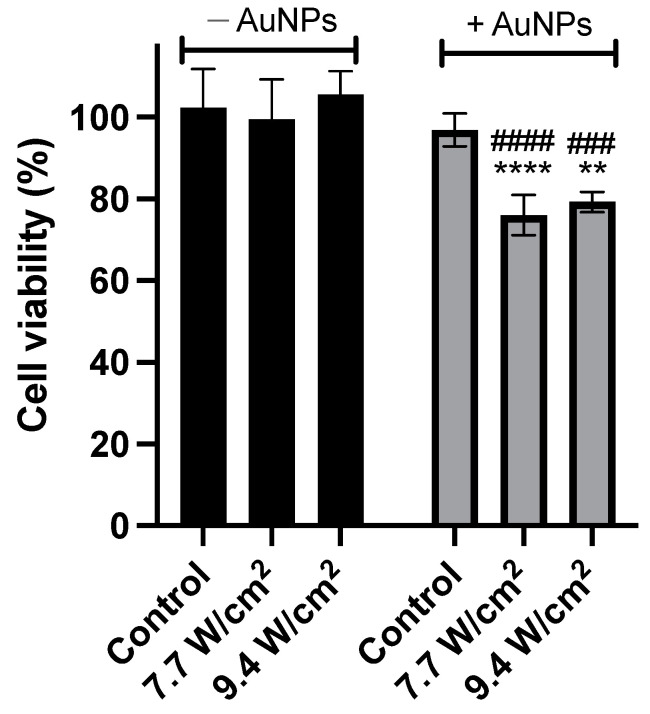
Cell viability (%) of PC-3 cells irradiated with laser at different irradiances and durations in the absence (dark grey) or presence (light grey) of AuNPs at 200 µM, and without laser irradiation as controls. The results represent the mean ± SD, n ≥ 3 (**** *p* < 0.0001, ** *p* < 0.0021, vs. respective control (control −AuNPs or control +AuNPs); ^####^ *p* < 0.0001, ^###^ *p* < 0.0002, vs. the same irradiance without AuNPs).

This test showed that for cells in the presence of a complete medium, the use of laser for both irradiation conditions did not significantly affect cell viability, suggesting the safety of the laser. However, when cells were incubated with AuNPs at the defined concentration, a significant reduction in cell viability was observed after irradiation when compared to non-irradiated samples. No significant differences were observed between irradiating cells incubated with AuNPs at an irradiance of 7.7 W/cm^2^ or 9.4 W/cm^2^, with a reduction in cell viability of 23.5% ± 4.5% and 26.3% ± 2.0%, respectively. The data is shown in Figure 7.

### 2.5. Preliminary In Vivo Safety Assessment

**Figure 8:**
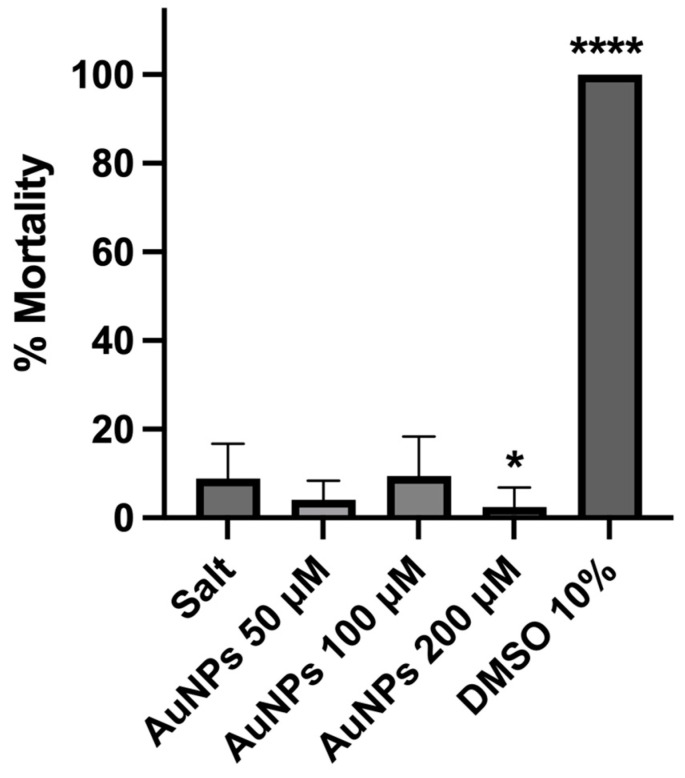
Mortality (%) of *Artemia salina* incubated for 24 h with only *artemia* salt medium (control), with different concentrations of AuNPs (50, 100 and 200 μM), and with 10% of DMSO (positive control). The results represent the mean ± SD, n ≥ 3 (**** *p* < 0.0001, * *p* < 0.0332, vs. salt).

The safety of the AuNPs was determined in vivo using the *Arteria salina* lethality assay. AuNPs were tested at 3 different concentrations: 50, 100 and 200 μM. As shown in Figure 8, all AuNPs did not induce in vivo toxicity in contrast with the positive control (10% of dimethyl sulfoxide, DMSO), therefore confirming the safety of the synthesized AuNPs.

## 3. Discussion

This work explores the use of PTT combined with AuNPs to selectively promote tumour ablation. AuNPs are of particular interest to improve the hyperthermia of tumour cells as they present tuneable properties, strong absorption in SPR and they can be coated and functionalized. The synthesis of AuNPs can be carried out by physical, chemical, and electrochemical methods [38,42], with the chemical method being the oldest and most widely used due to the homogeneity of formulations obtained [37]. The chosen synthesis method impacts the physicochemical, electrical and optical characteristics of AuNPs and, therefore, their biological function [43,44]. So, it is possible to produce AuNPs with the desired size, shape, distribution, surface area and surface charge for their application when prepared under controlled synthesis conditions [44,45]. In general, chemical synthesis takes advantage of reducing agents to reduce the metal salt and it can sometimes include the use of capping agents to stabilize and prevent further aggregation [37,43]. Various chemical synthesis methods have been described, such as the Turkevich method, the seed growth method, the Brust–Schiffrin method, etc. [40]. However, the most frequently-used ones require the use of harmful reagents such as CTAB, which is a flammable and cytotoxic substance by itself [46,47,48]. For this reason, approaches using low toxic reagents have already been proposed, such as Rosmarinic Acid (RA), which is the main component of the plant extract obtained by *Plectranthus saccatus Benth* and has shown favourable results [49,50]. In the present work, AuNPs were synthesized using a mixture of reducing agents including RA as the reducing and stabilizing agent instead of CTAB. Resultant AuNPs were characterized in terms of physicochemical characteristics and safety, as well as in terms of in vitro efficacy.

First, AuNPs were successfully synthesized. During the syntheses, there was an observable change of the colour of the colloidal suspensions, suggestive of AuNPs’ formation [41]. Specifically, before reacting with the reducing agents, it presented a yellow colour, which changed after the reaction with reducing agents to a dark blue colour.

Since we intend to locally inject AuNPs into the tumour and then activate them with an external light source, more precisely at the NIR wavelength, their characteristics such as size, surface charge, shape or surface chemistry are crucial for how they interact with cells [51].

The size and shape of the AuNPs will have an impact on the physicochemical and optical properties required as well as the effectiveness of cellular uptake, internalization, intracellular localization, cytotoxicity, biodistribution and, consequently, the different biological reactions that can occur [38,52,53]. So, AuNPs must be large enough to be retained at the injection site (in the tumour) with minimal migration to non-targeted tissues and not too large to make internalization difficult [54,55,56]. On the other hand, they should delay their clearance by the immune system [55,57]. Smaller AuNPs have broader biodistribution than larger nanoparticles [57]. It is known that particles of 50 up to 400 nm are capable of being retained in the tumour [58,59]. Other groups have reported that particles with larger sizes (≥200 nm) [54] are more difficult to diffuse in the tumour mass. Finally, particles smaller than 20 nm can easily pass to other non-targeted organs, such as through the blood–brain barrier and the placental barrier [38,60].

Considering the previous issues, particles with sizes of 50 nm but less than 200 nm seem to be able to increase their accumulation at tumour sites, preventing their extravasation from the tumour site by fenestrated capillaries with defective vasculature [54,55,61]. Considering what was listed above, it is suggested that the sizes obtained are favourable for in situ application of the AuNPs once the AuNPs presented a mean size of 108 nm. Contrarily, if they were smaller, there was a higher risk of them entering the systemic circulation through leaky vasculature [59,62,63].

Also, with regard to the size of the AuNPs, it is important to ensure the homogeneity of the formulation [63]. In fact, a monodisperse population is more reliable in terms of predicting the in vitro and in vivo behaviour of AuNPs [64]. However, narrow particle distribution is a limitation of the metallic NPs as their synthesis is generally not well controlled. The size distribution was measured by DLS. PdI is an autocorrelation that determines the degree of polydispersity of particles. PdI becomes a key factor to evaluate ranges from 0 (very homogenous particle population) to 1 (representation of a highly heterogenous population) [65]. Although there is no established consensus because it will depend on the material of the nanoparticle, according to ISO 22412:2017, samples with PdI < 0.4 are considered homogeneous, >0.4 less homogeneous, and ≈1 completely heterogeneous [66]. Nevertheless, values around <0.3 are considered optimal by some authors [63]. We obtained a mean value in line with a homogeneous particle population of approximately 0.12, indicating high monodispersity. Given the particles synthesized in this work, homogeneous populations with optimal values were obtained, i.e., the synthesis was well controlled.

Another important point is the surface charge of nanoparticles which might be predictive of the stability, toxicity, permeability and in vivo circulation time [41,65]. The charge that particles have on the surface is screened by an increased concentration of oppositely charged ions near the surface of the NPs (which form a Stern layer) [67]. The electric potential at the boundary of the Stern layer is the ZP of the particles and typically assumes values from −100 up to +100 mV [65]. In general, nanoparticles with ZP values greater than +30 mV or less than −30 mV have high degrees of physical stability. ZP can be determined through electrophoretic light scattering (ELS), in which the velocity of particles is analysed when they are submitted to an electric field [68]. In vivo, neutral particles undergo less opsonization than any of the charged particles, so they are preferred over the others [69]. Positively charged AuNPs are associated with greater cellular uptake, even in non-target tissues, due to their interaction with negatively-charged cell membranes [70]. Thus, cationic NPs are associated with high toxicity and lower stability than anionic NPs, leading to a preference for the use of the latter, which also has the advantage of accumulating in tumours as demonstrated in some studies [71,72]. When we analysed our synthesized AuNPs, they exhibited a negative surface charge, a suitable characteristic for accumulating at tumour sites as demonstrated elsewhere [71,73].

The biological interactions of AuNPs and their optical properties are also influenced by their morphology [74]. Most of our AuNPs were spherical in shape. The spherical shape is used to increase stability as it maximizes contact surface [75], allowing for easier internalization into the cell [76] and excretion, reducing possible toxicity [75].

Due to the possible NIR light absorption, there is a major interest in using AuNPs in PTT for cancer therapy, so the amount of heat generated is critical for effective cancer treatment. Thus, the absorption spectrum is a very important factor in the characterization of AuNPs as the absorption of light by NPs at certain wavelengths is essential for the conversion of light energy into thermal energy [77,78] to induce hyperthermia [79,80].

A beam of light from the instrument interacts with the sample. Energy is absorbed when the wavelength corresponds to the energy level that promotes an electron to a higher molecular orbital. Thus, the wavelength is determined by the maximum absorption peak of the sample [81]. The bandwidth of the spectrum, magnitude and wavelength peak of the plasmon resonance linked to the NP depends on the composition of the material, environment, shape and size of the material [82,83]. The AuNPs synthesized in this work present a broad band starting from 500 nm up to 900 nm. The laser used emits a radiation at 808 nm, and at this wavelength, the absorbance of AuNPs was greater than 0.5. Moreover, the synthesized AuNPs have an improved SPR band (627 nm) compared to the formulation previously presented by Amaral et al. 2021, developed for anaplastic thyroid carcinoma (551 nm) [48].

Safety and efficacy are very important aspects in any formulation development. Before activation, AuNPs should be stable and safe. After activation of the AuNPs with the laser, they must be cytotoxic for cancer cells.

The safety of AuNPs was tested in vitro on PCA cells by MTT assay. This assay is a widely used standard method that evaluates mitochondrial activity to draw conclusions about cytotoxicity [84]. In this study, it was observed that cell viability was maintained according to ISO 10993-5:2009(E) standards with a cell viability of over 95%, confirming the safety after 24 h of incubation at the tested concentrations [83,85,86]. In the past, similar results were obtained with human keratinocyte (HaCat) cell line but also in cancer cell lines such as A375, MCF-7, Bx-PC3 and U251 [8,58]. Moreover, Lopes et al. [58] reported the safety of AuNPs, showing that although presenting different sizes, AuNPs can be considered safe in vitro for different cell lines not only at 200 µM but also at much higher concentrations, up 600 µM. Another study has demonstrated the safety of larger AuNPs than the ones herein developed, showing once more that AuNPs at concentrations below 300 µM are considered safe for non-cancerous cells (HaCat) [87,88]. Moreover, and although at much lower concentrations, Leonavičienė et al. demonstrated not only the safety of AuNPs of smaller sizes, but also the efficacy on swelling reduction in collagen-induced arthritis following intra-articular administration in the joints of rats with collagen-induced arthritis [89]. In addition, the effects of AuNPs on other widely used human PCA cell lines have already been tested. Composite formulations of AuNPs were reported in DU145 cell lines with a reduction of less than 10% in cell viability [90]. Moreover, the viability of PCA cell lines (PC-3, DU145 and LNCaP) treated with AuNPs and ionizing radiation was assessed by Soares S. et al., with similar results between PC-3 and other PCA cell lines [91].

A preliminary in vivo study may provide an additional basis for evaluating their safety [92]. The *Artemia salina* mortality assay has been widely applied for studying NP toxicity [93]. The results obtained in the present work supported the safety of AuNPs at the three concentrations tested, 50, 100 and 200 μM, using the *Artemia salina* assay.

The efficacy studies were assessed by incorporating the AuNPs into agar phantoms (which mimic biological tissue [94]) and applying NIR laser irradiation (808 nm) to evaluate the temperature increase over 10 min. An exponential temperature increase pattern was initially observed, followed by a plateau-like phase in all the samples (50, 100 and 200 μM) and the positive control. According to the results obtained, we observed that the higher the concentration of AuNPs, the greater the temperature increase over the same period. This study also suggested that the AuNPs generated more heat than the positive control at the same irradiation exposure, confirming the potential of those AuNPs for the treatment of localized PCA. Results were in agreement with a previous study in breast cancer [95].

Finally, the efficacy of the synthesized AuNPs at 200 μM was tested with and without laser irradiation on PC-3 cell line. The use of the laser alone, without the presence of AuNPs, proved to be safe in terms of cell viability, as had already been published by other groups [96]. In addition, AuNPs incubated for 4 h in PC-3 cells, but without laser irradiation, also proved to be safe. This suggests that the isolated components are safe. On the other hand, in the combination, there was a decrease in cell viability, a very promising value for further tests. There were no significant differences in terms of reduced cell viability for the groups treated under the different conditions (7.7 W/cm^2^ and 9.4 W/cm^2^), although in both conditions there were significant differences compared to the untreated cells (control). More tests should be carried out to better understand the interaction of tumour cells and AuNPs. As example, in colon cancer cells [97], the increase in incubation time after irradiation with an 808 nm laser is accompanied by an increase in cell death, although with different ratios of apoptosis and necrosis. Consequently, as the fluence rate (W/cm^2^) increases, the percentage of apoptosis decreases for all incubation times after irradiation. The percentages of cell death by apoptosis are maximum at the moment immediately before the necrotic threshold fluence rate (50% necrotic pathway). Therefore, the apoptotic threshold (50% apoptosis) takes place at a lower fluence rate [97]. It has been shown that temperatures between 42 °C and 47 °C led to apoptosis [98,99]. In leukaemia cells, after 1 h at 42 °C–43 °C pre-apoptotic cells appear, which is interesting since it is the temperature that the body supports systemically in extreme fever situations [99].

## 4. Materials and Methods

### 4.1. Materials

#### 4.1.1. Reagents

Gold (III) chloride trihydrate (HAuCl_4_·3 H_2_O), L-Ascorbic acid (L-AA), silver nitrate (AgNO_3_), rosmarinic acid (RA), DMSO and phosphate-buffered saline (pH 7.4, PBS) were all supplied by Sigma-Aldrich (St. Louis, MO, USA). The water used was purified through a Millipore system (Millipore, Burlington, MA, USA). All the remaining reagents were of analytical grade.

#### 4.1.2. Cell Lines and Cell Culture

In vitro safety and efficacy of AuNPs were assessed in a PCA cell line (human PC-3 cells) (ATCC^®^CRL-1435™). Cells were maintained in Dulbecco’s modified Eagle’s medium (DMEM), with high glucose (4500 mg/mL) supplemented with 10% of fetal bovine serum (FBS), 100 IU/mL of penicillin and 100 µg/mL of streptomycin. Cells were kept in an incubator (NuAire NU-5500E, NuAire, Plymouth, MN, USA) at 37 °C and 5% CO_2_ atmosphere, and every two days the cell medium was changed when a confluence of 80% was reached.

#### 4.1.3. *Artemia salina* Culture

*Artemia salina* eggs and artificial sea water salt for *artemia* growth were purchased from JBL GmbH and Co., KG (Neuhofen, Germany). Artificial seawater was prepared by dissolving commercial seawater salt in tap water following the product instructions. *Artemia salina* eggs were added and left to hatch for 48 h and kept at a temperature from 25 to 30 °C, with continuous illumination and aeration.

### 4.2. Methods

#### 4.2.1. Synthesis of AuNPs

AuNPs were synthesized using a modified version of a synthetic method previously reported [41], replacing potentially toxic reagents such as CTAB with more friendly reagents such as RA. Briefly, the AuNPs were prepared based on a mixture of reducing agents (RA, L-AA (2 mM) and AgNO_3_ (1 mM)) with HAuCl_4_·3H_2_O (0.5 mM) at room temperature (RT), under magnetic stirring (800 rpm) (Heidolph MR3001, Heidolph Instruments, Schwabach, Germany) for 15 min. Afterwards, the AuNPs were stored at 4 °C, protected from light. The particles were centrifuged at 1520× *g* for 20 min (HERMLE Z 233 M, Hermle LaborTechnik GmbH, Wehingen, Germany) to remove unreacted reagents, and the pellets were resuspended in MilliQ water. Next, the colloidal AuNPs were stored at 4 °C, protected from light, until being used [41].

#### 4.2.2. Physicochemical Characterization

AuNPs were characterized in terms of mean particle size and PdI through DLS (Zetasizer Nano S; Malvern Instruments, Malvern, UK) using a constant scattering angle of 173°. AuNPs were diluted in MilliQ water in a ratio of 60:940 (*v*/*v*) for these measurements. These samples were also submitted to ELS, to determine the ZP (Zetasizer Nano S; Malvern Instruments, Malvern, UK). The samples were diluted in PBS pH 7.4 (1:10, *v*/*v*). Both assays were made in 3 series of 11 measurements of each analysed sample and at a constant temperature of 25 °C. Moreover, the maximum absorbance peak was evaluated from an absorbance spectrum, in a range from 400 to 1000 nm, by UV-visible spectroscopy (Shimadzu UV-21 160A; Shimadzu Europa GmbH, Duisburg, Germany). Samples were analysed in water.

To study the morphology of the synthesized AuNPs, AFM analysis was used. Forty microliters of the aqueous suspension of AuNPs were placed on a freshly cleaved mica surface and allowed to attach and left to dry for 1 h at RT. After that time, it was dried with nitrogen gas to remove any water residue. Images were acquired at a scan rate of 1 Hertz using the peak force and the ScanAsyst modes in a Multimode 8 HR coupled to a NanoScope V Controller (Bruker, Coventry, UK). The tip model used was the ScanAsyst-air 0.4 N/m (Bruker, Coventry, UK). Images were prepared using the NanoScope V 1.8 imaging software.

#### 4.2.3. Efficacy Assessment in a Phantom Model

**Figure 9:**
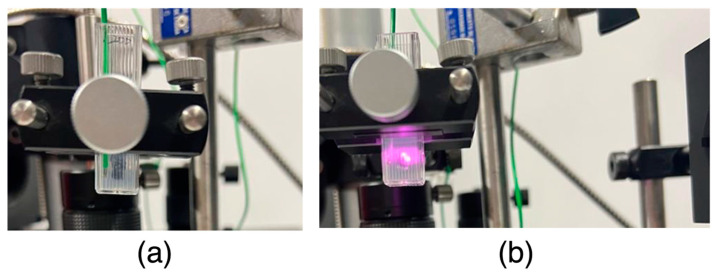
(**a**) Aligning the centre of the phantom with the laser and thermocouple next to the wall; (**b**) phantom irradiated with NIR laser in the centre of the well containing the material to be evaluated.

To study the thermal activity of AuNPs, an in vitro assay using a phantom model was performed, as represented in Figure 9. First, 1% (*w*/*v*) agar solution was prepared by dissolving the agar in water on a hot plate under magnetic stirring (Heidolph magnetic stirring hotplate MR 3001, Heidolph Instruments, Schwabach, Germany). Then, 1 mL of this solution was pipetted into each polystyrene cuvette and samples were left at 2 °C. When completely jellified, a small well was made in the centre of each cuvette with a pipette tip and 20 µL of each AuNPs suspension at the concentrations to be tested (50, 100 and 200 µM) were placed in this space. The negative control phantoms were made only by agar. For the positive control, the same procedure was followed, but instead of placing the AuNPs, a small black plasticine ball (diameter of 0.40 ± 0.05 cm) was inserted in the well of each phantom. To finalize the production of the phantoms, 1 mL of agar solution was then pipetted into each cuvette to cover the wells that had been previously filled and allowed to completely jellify at 2 °C. Once the prepared phantoms were assembled in the irradiation setup, a thermocouple (Fluke 52 K/J thermometer, Everett, WA, USA) was immersed in the phantom at same level of the AuNPs suspensions but in the front left vertices of the cuvette. The temperature variation was determined, i.e., before and after irradiation. A FC-808-2W Fiber Coupled Laser System (Frankfurt Laser Company, Friedrichsdorf, Hessen Germany) coupled to an FPYL-COL-X collimator (Frankfurt Laser Company, Friedrichsdorf, Hessen Germany) emitting at a wavelength of 808 nm (in the NIR range) was used. The laser beam was collimated, centred, and aligned to irradiate the centre of the cuvette containing the testing samples. The laser, with irradiance of 7.7 W/cm^2^, irradiated each phantom up to 10 min delivering an energy density of 47.77 J/mm^2^. The irradiation dose was selected based on previous works [85].

#### 4.2.4. In Vitro Safety Assessment without Laser Activation

The safety of AuNPs was evaluated in PC-3 cells. Cells were seeded in 96-well plates (200 µL/well) at 5 × 10^4^ cell/mL and allowed to adhere overnight in the culture conditions specified above. The next day, all the medium was removed and the cells were incubated in the same culture conditions with the AuNPs in the complete medium at a concentration of 50, 100 and 200 µM of Au content. After 24 h, the medium was removed, and cell viability was assessed by the MTT method. MTT is a standard technique widely used and assesses mitochondrial activity [84]. Cells were subsequently washed twice with PBS and 50 μL of MTT in an incomplete medium (0.5 mg/mL) were added to each well and incubated at 37 °C in a 5% CO_2_ atmosphere for 3 h. The formazan crystals formed after MTT reduction were solubilized by adding 100 μL of DMSO to each well, and absorbance was measured at 570 nm using a BioTek Elx800 absorbance microplate reader (BioTek Instruments, Inc., Winooski, VT, USA). The percentage of viable cells was determined according to Equation (1) with *OD_t_* being the optical density of cells incubated with the tested formulations and *OD_c_* representing the optical density of control cells corresponding to 100% cell viability.

(1) $$Cell\;viability(\%)=\frac{{OD}_{t}}{{OD}_{c}}\times 100$$

#### 4.2.5. In Vitro Efficacy Assessment Using Laser Activation

**Figure 10:**
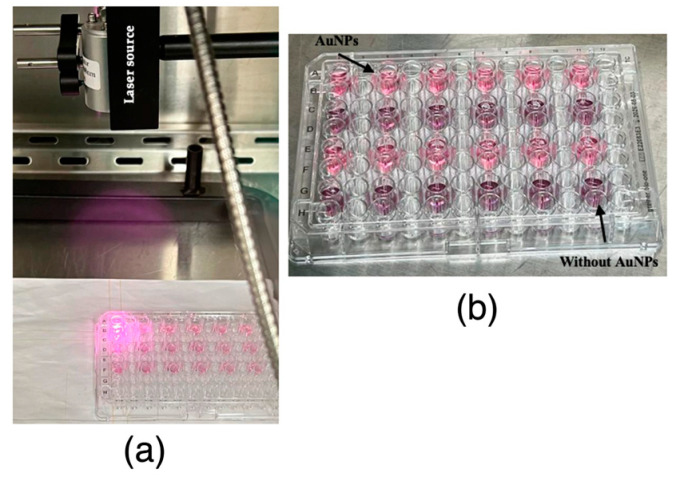
(**a**) Irradiation of the centre of each well to evaluate the AuNPs’ efficacy; (**b**) 96-well plate with wells ready for irradiation with and without AuNPs.

Human PC-3 cells were seeded in 96-well plates at a concentration of 5 × 10^4^ cell/mL and allowed to adhere overnight in the culture conditions specified above. The next day, all the medium was removed and the cells were incubated in the same culture conditions with formulation (200 µM) for 4 h (Figure 10). After this time period, the medium was removed and replaced with fresh medium in order to remove the unbound AuNPs, and the wells were irradiated with a NIR laser FC-808-2W Fiber Coupled Laser System (Frankfurt Laser Company, Friedrichsdorf, Hessen Germany) coupled to an FPYL-COL-X collimator (Frankfurt Laser Company, Friedrichsdorf, Hessen Germany) emitting at a wavelength of 808 nm with an irradiance of 7.7 W/cm^2^ (energy density of 14.33 J/mm^2^) and 9.4 W/cm^2^ (energy density of 29.03 J/mm^2^) for 3 min, centred in the middle of each well. Parallelly, a second plate at the same conditions with AuNPs was maintained without being irradiated by laser. In order to assess possible laser toxicity, cells without AuNPs were also irradiated.

After twenty-four hours, cells were washed twice with PBS 7.4, and 50 µL of MTT (Sigma-Aldrich, St. Louis, MO, USA) in an incomplete medium (0.5 mg/mL) was added. The cells were left to incubate with the reagent for 4 h to form the formazan crystals that were solubilized with DMSO. A BioTekTM EL × 800TM Absorbance Microplate Reader (Fisher Scientific, Hampton, NH, USA) at 570 nm was used for absorbance measurement and to perform the MTT assay. As with the previous assay, control wells, with only cells incubated with a complete medium, corresponded to 100% viability. Viability was calculated according to Equation (1).

#### 4.2.6. In Vivo Safety Assessment

Incubation with the AuNPs was performed and three concentrations of each AuNP batch were tested (50, 100 and 200 µM). Aeration was stopped before transferring 900 µL of artificial seawater containing 10–15 nauplii to each well of a 24-well plate to allow the dead *artemia* and eggs to settle to the bottom of the growth vessel, or to float at the artificial seawater–air interface. Next, 100 µL of different initial solutions were placed in the wells already with nauplii: artificial sea water only (negative control), 100% DMSO (positive control) and AuNP suspension from all concentrations. They were exposed to these solutions for 24 h under the same conditions as for growth, but without aeration. At the end of this time, the number of dead nauplii were counted. At the end, to kill the remaining nauplii, 100 µL of DMSO 100% were added and after 24 h, the total number of *Artemia salina* were counted and the mortality rate was established according to the equation bellow, where Dead_24h_ was the number of dead nauplii after 24 h of incubation with the samples and Dead_Total_ was the total number of nauplii in each well according to Equation (2). All samples were tested with four replicates.

(2) $$Mortality\left(\%\right)=\frac{D{ead}_{24h}}{{Dead}_{Total}}\times 100$$

#### 4.2.7. Statistical Analysis

All results presented are expressed as mean ± standard deviation (SD) for the respective n. A one-way ANOVA followed by Tukey’s multiple comparisons test was performed to analyse the results from the safety assays using *Artemia salina*, thermal activation using phantoms, and in vitro safety of AuNPs without laser irradiation. In addition, in vitro efficacy assessment was analysed by a two-way ANOVA followed by Sidak’s multiple comparisons test and Dunnett’s multiple comparisons test. A *p*-value < 0.05 was considered as statistically significant. All statistical analyses were performed using the GraphPad Prism software, version 9.1.2 (GraphPad Software, San Diego, CA, USA).

## 5. Conclusions

PCA is one of the most common cancers among men worldwide. Common treatments can have side effects that may impact a patient’s quality of life, including urinary incontinence, erectile dysfunction, fatigue, bowel problems and changes in hormone levels. It is essential to search for new, effective and promising treatments for PCA with the least adverse effects possible.

PTT using AuNPs as PTAs is a promising approach since it is minimally invasive and it is confined to the tumour site.

In this work, AuNPs have been successfully prepared and demonstrated an adequate size (around 100 nm and less than 200 nm) and a spherical shape for potential intratumoural administration, with optimal dispersibility (PdI less than 0.12).

These AuNPs have been shown to be safe in PCA cells, as well as in preliminary in vivo tests in the brine shrimp model. On the other hand, AuNPs were effective at improving the local temperature after irradiation at the required temperature to promote tumour cell death. As the concentration of AuNPs increases, the temperature increase is also greater over the same time interval. After being irradiated, the AuNPs were shown to interfere with the cell viability of PCA cells.

In conclusion, the overall results obtained are very promising and show potential for using the AuNPs produced as part of a PTT system.

## Figures and Tables

**Table 1:** AuNPs’ physicochemical characterization. Values are represented as mean ± SD, with n ≥ 3.

Mean Size (nm) ± SD	Mean PdI ± SD	Mean ZP (mV) ± SD	Maximum Absorbance Peak (nm)	Absorbance at 808 nm
108 ± 15	0.119 ± 0.014	−18 ± 2	627	0.579

## Data Availability

Data is contained within the article.
